# Potential Mechanisms for IgG4 Inhibition of Immediate Hypersensitivity Reactions

**DOI:** 10.1007/s11882-016-0600-2

**Published:** 2016-02-18

**Authors:** Louisa K. James, Stephen J. Till

**Affiliations:** Randall Division of Cell and Molecular Biophysics and MRC and Asthma UK Centre for Allergic Mechanisms of Asthma, King’s College London, London, SE1 1UL UK; Division of Asthma, Allergy and Lung Biology, King’s College London and Department of Allergy, Guy’s and St. Thomas’ NHS Foundation Trust, London, SE1 9RT UK

**Keywords:** IgG4, Hypersensitivity, Blocking antibodies, Allergy, Allergen immunotherapy, IgE

## Abstract

IgG4 is the least abundant IgG subclass in human serum, representing less than 5 % of all IgG. Increases in IgG4 occur following chronic exposure to antigen and are generally associated with states of immune tolerance. In line with this, IgG4 is regarded as an anti-inflammatory antibody with a limited ability to elicit effective immune responses. Furthermore, IgG4 attenuates allergic responses by inhibiting the activity of IgE. The mechanism by which IgG4 inhibits IgE-mediated hypersensitivity has been investigated using a variety of model systems leading to two proposed mechanisms. First by sequestering antigen, IgG4 can function as a blocking antibody, preventing cross-linking of receptor bound IgE. Second IgG4 has been proposed to co-stimulate the inhibitory IgG receptor FcγRIIb, which can negatively regulate FcεRI signaling and in turn inhibit effector cell activation. Recent advances in our understanding of the structural features of human IgG4 have shed light on the unique functional and immunologic properties of IgG4. The aim of this review is to evaluate our current understanding of IgG4 biology and reassess the mechanisms by which IgG4 functions to inhibit IgE-mediated allergic responses.

## Introduction

The identification of four distinct subclasses of human IgG was first described during the 1960s, when they were designated as IgG1, IgG2, IgG3 and IgG4, based on their relative concentrations in human serum [1–3]. IgG4 was notable as the least abundant IgG subclass with an average serum concentration of 0.4 mg/ml, compared to 8 mg/ml for IgG1. In addition, unlike the other IgG subclasses, IgG4 is unable to fix complement or precipitate antigens. IgG4 is comprised of two identical 50-kDa heavy chains each consisting of four distinct immunoglobulin domains (VH, CH1, CH2 and CH3) and two identical 25-kDa light chains each consisting of two immunoglobulin domains (VL and CL). The 12 amino acid hinge region between CH1 and CH2 provides mobility of the variable Fab regions in relation to the Fc region, facilitating binding of antigen. All of the IgG subclasses share a high degree of sequence homology, but key differences in the hinge region and Cγ2 domain give rise to important variations in effector function.

IgG antibodies interact with immune cells through binding to Fcγ receptors expressed on cell surfaces [4•]. With the exception of the neonatal Fcγ receptor (FcγRn), which binds at the Cγ2-Cγ3 interface and functions primarily to transport IgG across placental and mucosal surfaces, all Fcγ receptors bind at the N-terminus of Cγ2. There are seven Fcγ receptors in humans (Table 1); FcγRI has the highest overall affinity for IgG and can bind monomeric antibody. This high affinity means that FcγRI is saturated with IgG, although similar to the high affinity IgE receptor FcεRI, signaling only occurs following antigen cross-linking [5]. The other Fcγ receptors generally have lower affinity (100–1000-fold less) for IgG subclasses and hence bind only to immune complexes and not to monomeric antibody.

**Table 1:** Cellular expression and relative binding affinities of human Fcγ receptors

Receptor	Cellular expression	IgG1	IgG2	IgG3	IgG4
FcγRI/CD64	Monocytes, macrophages, DC, neutrophils^a^, mast cells^a^	++++	–	++++	++++
FcγRIIa/CD32a	Monocytes, macrophages, DC, basophils, mast cells, eosinophils, platelets	+++	++	+++	++
FcγRIIb/CD32b	B cells, DC, basophils, neutrophils subsets of monocytes and macrophages	+	–	++	+
FcγRIIc/CD32c	NK cells, monocytes, macrophages and neutrophils	+	–	++	+
FcγRIIIa/CD16a	Natural killer (NK) cells, monocytes and macrophages	+++	+/-	++++	+/-
FcγRIIIb/CD16b	Neutrophils subsets of basophils	+++	–	+++	–
FcγRn	Monocytes, macrophages, DC, neutrophils, epithelial cells, endothelial cells	++++	++++	++++	++++

There are two polymorphic variants of FcγRIIa (131H and 131R) with similar binding properties. FcγRIIIa has two polymorphic variants (158V and 158F). IgG2 and IgG4 bind only to FcγRIIIa^158V^ and only as immune complexes, whereas IgG1 and IgG3 bind to both variants with high affinity [6]. IgG binding to FcγRn only occurs at pH < 6.5 but it binds to all IgG subclasses [60]

^a^Induced following activation

IgG4 binds to all of the Fcγ receptors with the exception of FcγRIIIb (Table 1), which contains a membrane anchored GPI domain and thus cannot induce intracellular signaling [6]. FcγRI and FcγRIIIa both associate with the common γ chain, which contains an intracellular immunoreceptor tyrosine-based activation motif (ITAM), driving cellular activation upon receptor engagement. FcγRIIa and FcγRIIc are single-chain activating receptors and also possess an ITAM in their intracellular domains. Signaling through activated Fcγ receptors promotes a range of effector functions including internalization of Fc-bound immune complexes, enhanced antigen-presentation, antibody-dependent cell-mediated cytotoxicity (ADCC) and cellular activation [7•, 8]. In contrast, FcγRIIb is a single-chain inhibitory receptor, the only inhibitory Fcγ receptor in humans, possessing an immunoreceptor tyrosine-based inhibition motif (ITIM) in the intracellular domain. IgG4 is the only IgG subclass that can bind with equal affinity to both FcγRIIb and the activating receptors. Co-ligation of FcγRIIb with activating Fcγ receptors results in inhibition of effector cell responses [9]. FcγRIIb also plays an important role in regulation of B cell activity and plasma cell survival [10].

The crystal structure of human IgG4 Fc was first solved in 1997 in complex with an IgM rheumatoid factor [11]. More recently, a higher resolution structure of IgG4-Fc was solved, providing further insights into the unique structural features of IgG4 [12••]. Notably, conformational differences in two key loop structures in the Cγ2 domain of IgG4 compared to IgG1 provide a structural basis for the lower binding affinity of IgG4 to some of the Fcγ receptors and the inability of IgG4 to bind the complement component C1q.

### Fab Arm Exchange

Cysteine residues in the hinge region of IgG4 result in intra-heavy chain disulphide bonds, as opposed to the inter-heavy chain bonds present in the other IgG subclasses. In addition, a key amino acid substitution in the Cγ3:Cγ3 interface weakens the domain interactions. Under reducing conditions, the combined effect allows dissociation of the two heavy chains of human IgG4 into half-molecules [13]. Re-association of half-molecules originating from different IgG4 antibodies results in ‘bi-specific’ monovalent antibodies. IgG4 antibodies that have undergone this process are consequently unable to undergo antigen cross-linking to form immune complexes. Analysis of serum from healthy human subjects revealed that 20–30 % of monomeric IgG4 contained both κ and λ light chains within the same molecule, demonstrating that Fab arm exchange occurs in vivo in a substantial fraction of IgG4 [14•].

## IgG4 Production

Naïve B cells express IgM as a monomeric membrane-bound B cell receptor (BCR). Activation of naïve B cells through the BCR can lead to rearrangement of the immunoglobulin heavy chain locus through class switch recombination. This results in the expression of a different ‘switched’ isotype (IgG, IgA or IgE), dependent on additional signals provided by cytokines. Class switch recombination to IgG4 depends on the production of the Th2 cytokines IL-4 and IL-13, along with ligation of CD40 [15, 16]. These same signals (IL-4 plus CD40L) classically drive class switch recombination to IgE, the primary effector antibody involved in allergic disease [17]. There is a clear biological relationship between IgG4 and IgE production, although the molecular mechanisms that dictate recombination to IgG4 versus IgE have yet to be fully elucidated [18]. The addition of IL-10 [19] or IL-21 [20] to in vitro cultures can enhance or suppress the production of both IgG4 and IgE depending on the conditions under which B cells are stimulated. Although IL-10-producing B cells are reported to produce increased amounts of IgG4 in culture compared to IL-10^−^ B cells [21], IgG4^+^ B cells express equivalent amounts of IL-10 to their IgG1^+^ counterparts [22••]. This suggests that while IL-10 can influence the production of IgG4, IgG4^+^ B cells themselves are not the major source of this cytokine. Circulating IgG4^+^ B cells from healthy individuals have a similar but distinct phenotype to IgG1^+^ B cells: they lack surface IgD but express CD27, consistent with the phenotype of class-switched memory B cells [22••]. Expression of the complement receptor CD21 (CR2) is lower on IgG4^+^ B cells compared to IgG1^+^ B cells from the same individuals. CD21 forms part of the B cell receptor-signaling complex along with CD19 and CD81 and also has an important role in internalization of immune complexes [23]. Hence, lower expression of CD21 may result in reduced responsiveness to antigen and/or immune complexes. Furthermore, Lighaam and colleagues [22••] reported increased expression of the IgE receptor, CD23 (FcεRII) on IgG4^+^ versus IgG1^+^ B cells. CD23 expression is up-regulated by IL-4, an important switch factor for IgG4, again highlighting the close association between IgG4 and IgE.

### IgG4 and the Modified Th2 Response

An important feature of IgG4 production is the association with high-dose chronic antigen exposure. The production of allergen-specific IgG4 is linked to the ‘modified Th2 hypothesis’, whereby an allergen-driven Th2 response in which IgG4 dominates and IgE is absent results in protection from immediate hypersensitivity [24]. This was first described by Platts-Mills and colleagues who demonstrated that children exposed to high concentrations of the major cat allergen, Fel d 1, had high titers of Fel d 1-specific IgG4 and were clinically tolerant (ie not cat allergic) [25]. Similarly, high titers of allergen-specific IgG4 are observed following chronic exposure to other exogenous antigens including occupational allergens [26] and bee venom [27]. Thus, the balance between IgG4 and IgE production appears to critically influence the development of allergic hypersensitivity versus immune tolerance. Importantly, studies of IgG4-induced antibody responses in allergen immunotherapy (AIT) demonstrate that IgG4 is capable of directly inhibiting the activity of IgE.

### IgG4 and Allergen Immunotherapy

AIT is an effective treatment for IgE-mediated allergy and induces long-term clinical tolerance, associated with increases in IL-10-producing T regulatory cells and reductions in basophil reactivity [28]. The observation that IgG4 responses are associated with chronic high-dose natural allergen exposure is consistent with the effect of AIT—involving repeated administration of high-dose subcutaneous, sublingual or oral allergen over years—in inducing allergen-specific IgG4 [29–31]. The concept that treatment-induced IgG antibodies could provide protection from immediate hypersensitivity emerged early in the history of AIT. Pre-dating even the discovery of IgE, Cooke and Loveless demonstrated that post-immunotherapy serum could inhibit in vivo Prausnitz-Küstner reactions [32]. In 1982, Golden et al. demonstrated that titers of venom-specific IgG were significantly higher in patients who were successfully treated with venom immunotherapy, whereas treatment failure was associated with lower levels of specific IgG [33]. This led to the theory that specific IgG levels could correlate with the clinical response to treatment. During this time, it was established that despite initial low levels in serum, AIT appeared to stimulate marked increases in allergen-specific IgG4 [34]. However, data from certain clinical trials raised doubts regarding the relevance of allergen-specific IgG4 to the clinical benefit of AIT since high levels of allergen-specific IgG4 were associated with treatment failure rather than success [35].

### Inhibition of IgE Activity by IgG4

**Fig. 1:**
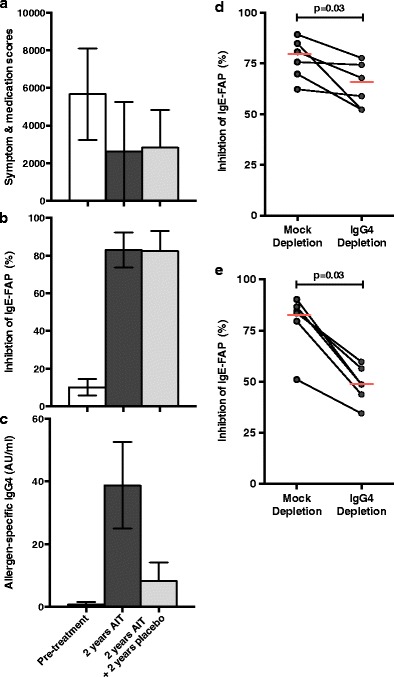
Continued reduction in symptom and medication scores 2 years after withdrawal of grass pollen immunotherapy is accompanied by persisting IgG4-mediated inhibitory activity against IgE-FAP. **a** Symptom and medication scores at baseline, after 2 years of allergen immunotherapy and 2 years after stopping treatment. **b** Inhibition of IgE-FAP by sera taken at baseline, after 2 years of allergen immunotherapy and 2 years after stopping treatment. **c** Serum grass pollen-specific IgG4 measured by ELISA at baseline, after 2 years of allergen immunotherapy and 2 years after stopping treatment. **d** Inhibition of IgE-FAP by mock-depleted or IgG4-depleted serum taken after 2 years of allergen immunotherapy. **e** Inhibition of IgE-FAP by mock-depleted or IgG4-depleted serum taken 2 years after stopping treatment. Figure reproduced with permission from Reference 38

Definitive evidence that IgG is able to inhibit IgE activity was provided by Van Neerven and colleagues who reported that AIT-induced IgG could inhibit IgE-facilitated allergen presentation (IgE-FAP) by B cells to T cells in vitro [36]. Later similar studies went on to show that this ‘blocking activity’ co-eluted with IgG4 [37, 38]. IgE-FAP depends on the binding of immune complexes formed of allergen and specific IgE to the low-affinity IgE receptor CD23 (FcεRII). Capture of IgE-allergen complexes by CD23-expressing antigen-presenting cells results in internalization and processing of the allergen-IgE-receptor complex with subsequent presentation of allergen-derived peptides to T cells [39]. The ability of IgG4 antibodies to inhibit this process relies entirely on the affinity, specificity and quantity of the blocking antibody, regardless of isotype or subclass [40, 41•]. Conventional subcutaneous immunotherapy induces significant increases in allergen-specific IgG4 6–8 weeks following the start of treatment [29]. This corresponds with the appearance of IgE-FAP inhibitory activity in serum but is preceded by increases in allergen-driven IL-10 production by peripheral blood mononuclear cells. The functional inhibitory activity of IgG4 appears to relate more closely to the clinical efficacy of AIT than absolute levels in serum, which may explain why levels of IgG4 often correlate poorly with clinical responses. In a randomized double-blind placebo controlled trial of subcutaneous grass pollen immunotherapy, continued clinical remission 2 years after treatment withdrawal was accompanied by persisting inhibitory antibody activity in serum against IgE-FAP [38]. Although the levels of serum allergen-specific IgG4 fell to near pretreatment values during the 2 years of treatment withdrawal, depletion experiments identified IgG4 as the main source of the continuing inhibitory activity. This study indicates that the activity but not quantity of IgG4 antibodies per se is the main determinant of clinically relevant IgE inhibition (Fig. 1).

In addition to inhibiting CD23-dependent IgE activity, IgG4 can also block the effects of IgE signaling through FcεR1, and in turn inhibit immediate hypersensitivity. For example, IgG4 purified from the serum of an immunotherapy-treated individual inhibited IgE-mediated basophil degranulation, which depends on cross-linking of high-affinity IgE receptor (FcεRI) -bound IgE [42]. The ability of IgG4 to inhibit FcεRI-dependent activity of IgE has been proposed to arise through two possible mechanisms; either through direct competition for allergen with receptor-bound IgE and/or through simultaneous binding of IgG4 to the inhibitory FcγRIIb. A high-affinity monoclonal IgG4 antibody specific for the grass pollen allergen Phl p 7 was able to inhibit IgE-mediated basophil degranulation in vitro [43]. In order to determine whether this activity was subclass-dependent, a panel of antibodies with identical specificity but different subclasses, namely IgG1, IgG2, IgG3, IgA1 and IgA2, was generated [41•]. The antibody specificity was found to be the critical determinant of the inhibitory activity, since each subclass was able to block basophil activation to an equal degree. Thus, any antibody isotype with sufficient affinity for allergen has the potential to effectively prevent cross-linking of IgE receptors though competition with IgE for allergen binding.

### IgG4 and FcγRIIb-Mediated Inhibition of IgE

Using a bi-specific antibody, Kepley and colleagues demonstrated that cross-linking of FcγRIIb and FcεRI inhibits IgE-mediated basophil activation [44]. The role of FcγRIIb in the inhibitory effect of AIT serum on basophil degranulation has been the subject of conflicting reports in the literature. In two independent studies using similar methodologies, the inhibitory activity of AIT serum on basophil activation was investigated by pre-incubation of basophils with antibodies to block human FcγRII. Whereas one study found that pre-incubation attenuated the inhibitory effect of AIT serum leading to increased basophil activation [45], the other study reported that FcγRII blockade had no effect on the inhibition of IgE-mediated basophil degranulation by AIT serum despite successful inhibition of IgG complex binding [46]. These two studies used different anti-FcγRII antibody clones, although neither was able to discriminate between the activating (FcγRIIa) and inhibitory (FcγRIIb) receptors expressed by human basophils. The direct effects of these antibodies on basophil function, e.g. activation by signaling through FcγRIIa, were not investigated but may have influenced the experimental outcomes. A further single report used two monoclonal antibodies selective for FcγRIIa and FcγRIIb, respectively, to assess the role of these receptors in IgG-mediated inhibition of basophil reactivity [47]. Intriguingly, the authors found that blocking either FcγRIIa or FcγRIIb attenuated the inhibitory activity of IgG from AIT serum, although as with previous studies, the direct effect of these monoclonal antibodies on basophil activation was not assessed [47].

The potential for interaction of allergen-specific IgG4 and FcγRIIb to result in effective inhibition of FcεRI signaling remains uncertain. Using surface plasmon resonance, Bruhns and colleagues found that IgG4 bound to FcγRIIb with moderate affinity (*K*_*A*_ 2 × 10^5^ M^−1^) and when IgG4 antibodies were aggregated as F(ab′)_2_ complexes, binding to FcγRIIb was detected on cell surfaces [6]. However, human IgG4 does not precipitate antigen and forms only small immune complexes compared to IgG1 [48], likely due to the dynamic process of Fab arm exchange [49]. This may have significant functional consequences for interactions between IgG4 and FcγRIIb, which have yet to be examined experimentally. Furthermore, the potential biological relevance of this pathway must also be considered, since although FcγRIIb is constitutively expressed by basophils, expression on mast cells is variable depending on tissue distribution; while peripheral blood [50], cord-blood derived [51] and synovial mast cells [52] all express FcγRIIb, expression has not been demonstrated on human skin mast cells [53] or intestinal mast cells from most individuals [54]. Therefore, at least in the skin and intestine, the potential relevance of IgG-mediated inhibition of mast cell activation through FcγRIIb pathways must be questioned.

## Conclusions

IgG4 is closely associated to the production of IgE and therefore has relevance to the study of allergic disease. Absolute levels of IgG4 often fail to correlate with clinical tolerance, although absolute levels of IgE are similarly poorly predictive of disease severity. Nonetheless, the biological activity of IgG4, in particular the potent inhibition of IgE-mediated basophil/mast cell activation and antigen presentation, suggest that this unique subclass is indeed relevant to disease expression. IgG4 has a long association with tolerance to aeroallergens, both in studies of allergen immunotherapy and the so-called modified Th2 response. An accumulating body of literature also supports a potential wider role for IgG4 in oral immunotherapy studies of food allergy [55] and also in natural tolerance to food allergens [56–59]. Experimental approaches need to be developed to address unresolved questions concerning IgG4 biology, such as identification of factors that regulate IgG4 versus IgE responses. Understanding the precise molecular determinants that control the fate of IgG4 versus IgE switching could highlight therapeutic targets for prevention of allergy and promotion of clinical tolerance.
